# Use of Google Analytics to Explore Dissemination Activities for an Online CKD Clinical Pathway: A Retrospective Study

**DOI:** 10.1177/20543581221097456

**Published:** 2022-05-11

**Authors:** Christy Chong, Michelle Smekal, Brenda Hemmelgarn, Meghan Elliott, Selina Allu, James Wick, Kerry McBrien, Wes Jackson, Aminu Bello, Kailash Jindal, Nairne Scott-Douglas, Braden Manns, Marcello Tonelli, Maoliosa Donald

**Affiliations:** 1 Department of Community Health Sciences, University of Calgary, AB, Canada; 2 Department of Medicine, University of Calgary, AB, Canada; 3 Department of Medicine, University of Alberta, Edmonton, Canada; 4 Department of Family Medicine, Cumming School of Medicine, University of Calgary, AB, Canada; 5 O’Brien Institute for Public Health, Cumming School of Medicine, University of Calgary, AB, Canada

**Keywords:** chronic kidney disease, clinical pathway, dissemination, Google Analytics, health informatics, Internet, primary care

## Abstract

**Background:**

Data on dissemination strategies that generate awareness of clinical pathways for kidney care are limited.

**Objective:**

This study reports the application of Google Analytics to describe the reach and use of the Chronic Kidney Disease Pathway (CKD-P) using a multi-faceted dissemination strategy.

**Design:**

The design of this study is a retrospective descriptive study.

**Setting:**

This study was conducted in Alberta, Canada.

**Patients:**

Individuals who accessed the CKD-P Web site between November 5, 2014, and May 31, 2019.

**Measurements:**

Dissemination activities included print, electronic, in-person meetings, and a laboratory prompt. We used Google Analytics over a 5-year period to evaluate the following CKD-P Web site user metrics: number of sessions, pageviews, visit duration, user path, and bounce rate (when an individual visits a single page of the Web site and leaves the Web site without interacting with additional pages).

**Methods:**

We plotted dissemination activities alongside Web site metrics using control charts and described the data using means and percentages. We performed chi-square test for trends to evaluate year-over-year usage.

**Results:**

There were 83 294 users, 90 805 sessions, and 231 684 pageviews. The overall bounce rate was 45.7%. Each user had an average of 1.5 sessions and a session duration of 2 minutes and 8 seconds. There was a significant positive trend for total annual users (*P* = .008), new users (*P* = .009), number of sessions (*P* = .006), and pageviews per day (*P* = .016).

**Limitations:**

We were unable to confirm if users were primary care providers and if word-of-mouth dissemination among providers/researchers drove people to use the CKD-P.

**Conclusions:**

Google Analytics was a useful and accessible tool for evaluating CKD-P reach and use trends. It was challenging to identify how individual dissemination activities contributed to CKD-P reach; however, repeated dissemination appeared to play a role in increasing CKD-P use.

**Trial registration:**

Not applicable—observational study design.

## Abrégé

**Contexte:**

Il existe peu de données sur les stratégies de diffusion pour sensibiliser les différents intervenants aux plans d’intervention en santé rénale.

**Objectif:**

Cette étude rend compte de l’utilization de Google Analytics pour décrire la portée et l’utilization d’un plan d’intervention en ligne pour l’insuffisance rénale chronique (CKD-P — *Chronic Kidney Disease Pathway*) à l’aide d’une stratégie de diffusion à plusieurs facettes.

**Conception de l’étude:**

Étude descriptive rétrospective.

**Sujets:**

Les individus ayant accédé au site Web CKD-P entre le 5 novembre 2014 et le 31 mai 2019.

**Mesures:**

Les activités de diffusion comprenaient des documents imprimés, des documents électroniques, des réunions en personne et un lien internet vers le site web de CKD-P lors de la transmission d’un résultat anormal de laboratoire. Nous avons utilisé Google Analytics pendant cinq ans pour évaluer les mesures suivantes pour les utilisateurs du site Web CKD-P: nombre de sessions, vues de page, durée des visites, chemin d’accès utilisateur et taux de rebond (lorsqu’une personne visite une seule page du site Web et le quitte sans interagir avec d’autres pages).

**Méthodologie:**

Les activités de diffusion et les paramètres du site Web sont présentés à l’aide de tableaux de contrôle, et les données à l’aide de moyennes et de pourcentages. Un test de Chi-Deux a servi à déterminer les tendances et évaluer l’utilization d’une année sur l’autre.

**Résultats:**

Nous avons répertorié 83 294 utilisateurs, 90 805 sessions et 231 684 pages consultées. Le taux de rebond global était de 45,7 %. En moyenne, chaque utilisateur comptait 1,5 session dont la durée moyenne était de 2 minutes 8 secondes. On a observé une tendance positive significative pour le nombre total annuel d’utilisateurs (*P* = 0,008), les nouveaux utilisateurs (*P* = 0,009), le nombre de sessions (*P* = 0,006) et les vues de pages par jour (*P* = 0,016).

**Limites:**

Il n’a pas été possible de confirmer si les utilisateurs étaient des fournisseurs de soins primaires et si la diffusion de bouche-à-oreille entre les fournisseurs/chercheurs avait amené les gens à utiliser CKD-P.

**Conclusion:**

Google Analytics s’est avéré un outil utile et accessible pour évaluer les tendances de portée et d’utilization de CKD-P. Il est difficile d’établir comment les activités de diffusion individuelles contribuent à la portée de CKD-P; la diffusion répétée semble néanmoins jouer un rôle dans l’augmentation de l’utilization de CKD-P.

**Enregistrement de l’essai:**

Sans objet — étude observationnelle

## What was known before

Online clinical pathway tools are used to translate clinical guidelines into practice. Within health care, Google Analytics has been used to monitor and describe knowledge dissemination strategies for guideline-based online clinical pathway tools. However, data on dissemination strategies that generate awareness of clinical pathways for kidney care are poorly understood.

## What this adds

A longitudinal multi-faceted dissemination strategy with repeated delivery of in-person activities increased Chronic Kidney Disease Pathway (CKD-P) awareness. The CKD-P visitors invested time into learning about current kidney guidelines. Google Analytics was a useful tool for evaluating pathway reach and use trends.

## Introduction

Chronic kidney disease (CKD), defined as an estimated glomerular filtration rate (eGFR) less than 60 mL/min/1.73 m^2^ for more than 3 months, affects approximately 12% of adults in Canada.^1^ The CKD is often accompanied by other comorbidities such as diabetes, hypertension, and cardiovascular disease,^2^ making medical management of this patient population challenging. However, timely diagnosis and early intervention can slow the progression of CKD and improve the care and outcomes of patients with CKD.^3^

Primary care providers play a pivotal role in the early diagnosis and management of patients with CKD^4^; thus, it is important that they are kept informed of evidence-based clinical practice guidelines to guide patient care. Clinical pathways are knowledge management tools that can be used to facilitate translation of evidence-based guidelines into practice and standardize patient care.^5^ They are effective knowledge translation tools that can improve communication between multidisciplinary fields, guide early career physicians, and improve the quality of patient care.^6^ Despite their potential advantages, there are limited clinical pathways available that are tailored for primary care providers who care for adults with CKD.^7^ Our team previously developed and implemented a Chronic Kidney Disease Pathway (CKD-P; www.ckdpathway.ca) that aimed to provide guidance and supporting information for primary care providers to aid in the diagnosis, management, and referral of adults with CKD.^8^ It was launched on November 5, 2014.^8^

Translating new knowledge into clinical settings requires effective dissemination, which is “the active approach of spreading evidence-based interventions to the target audience using planned strategies.”^9^ Common dissemination activities used by health researchers include news media (radio, television, newspapers), social media (Facebook, Twitter, blogs), policy briefs, one-on-one meetings, workshops, conferences, and seminars.^10^ A scoping review of current CKD clinical pathways found that, while most studies reported the purpose of the pathway tool, approaches to pathway dissemination and evaluation were not well described.^7^ Moreover, even though many evidence-based programs are intended to translate findings to a health care setting,^11^ less than 10% of time is spent on dissemination.^12^ Our team used a multi-faceted dissemination approach to bring awareness to health care professionals about the CKD-P.^8^ Currently, there is little information describing dissemination activities for online clinical decision-making tools, in addition to the lack of use of Google Analytics to report user behaviors and usage.^13^

Google Analytics is an informatics tool often used to assess Web site usage data.^14^ The information gathered from this tool can be leveraged to successfully optimize the usability and utility of Web sites.^14^ Within health care, Google Analytics can be used to monitor and describe knowledge dissemination strategies for guideline-based online clinical tools, such as the CKD-P. Limited studies have applied a rigorous, longitudinal approach to using Google Analytics as an analytics tool to evaluate kidney-related Web site dissemination and uptake.^13^ The aim of this study is to describe the reach and use of the CKD-P after the implementation of various dissemination activities using Google Analytics.

## Methods

### Study Overview

Over a 5-year period, a multi-faceted dissemination approach was used to increase awareness and interest about the CKD-P among health care professionals who care for adults with CKD (i.e. primary care physicians, pharmacists, nurses, nephrologists, academic institutions, policy makers, etc.).^8^ We used Google Analytics data to conduct a retrospective descriptive review of all dissemination activities implemented from November 5, 2014, to May 31, 2019. We assessed trends in Web site use using control charts; this is a tool that can be used to visualize how specific outcomes change over time.^15^ We plotted Google Analytics Web site metrics and dissemination activities in time order by 2-week increments. We included the means, positive 3 sigma limits from the mean, and negative 3 sigma limits from the mean in each control chart. We describe the reach of the CKD-P during this period when various types of dissemination activities (print, electronic, in-person meetings, laboratory prompt) were conducted.

### Web site Layout

The CKD-P tool is available on an open-access, public Web site (http://www.ckdpathway.ca/). It consists of the following pages: a *home* page containing an overview of the Web site; a *diagnose* page containing an interactive diagnostic tool for CKD; a *medical management* page containing information on recommended lifestyle changes, patient handouts, and drug therapy information; a *referral* page providing information on indications for referral; and a *resource* page containing additional information about CKD and links to external resources.

### Google Analytics Metrics and Data Analysis

We configured Google Analytics for the CKD-P Web site prior to Web site launch in November 2014. To evaluate CKD-P user traffic, we exported daily Web site traffic and user behavior data from November 5, 2014, to May 31, 2019. Metrics included details about the users, sessions, Web site pages, pageviews, and bounce rates (Box 1).^16^

**Box 1.** Google Analytics Metrics Collected.

Metrics	Definition
Total number of users	The number of users that visited any page on the Web site during the entire study period.
Number of new users	Users are tracked using cookies. When a new browser is used to access the Web site, this is considered a new user. New users also include previous users who deleted their cookies and then accessed the Web site.
Number of sessions	Interactions on the Web site. Every visit is considered a session.
Average number of sessions per user	The average number of times a user visited the Web site within the specified time period.
Number of pageviews	The number of times a user views a page. A new pageview is counted when a user refreshes the page or visits another page and comes back to it.
Average pages viewed per session	The total number of pages viewed over the total numbers of sessions.
Number of unique pageviews	The number of sessions where a page on the Web site is viewed at least once.
Average session duration	The average amount of time spent on the Web site per user. The end of a session occurs when a user leaves the Web site or there is at least 30 minutes of inactivity.
Average bounce rate	When an individual visits a single page of the Web site and leaves the Web site without interacting with additional pages.
Starting page	The first page users begin their Web site session.
First interaction page	The next page that users visit after leaving the starting page.
Second interaction page	The next page that users visit after leaving their first interaction page.

We tracked user behavior by analyzing the number of entrances, pageviews, unique pageviews, average session duration, percentage of exits, starting pages, interaction pages, and the bounce rate for each web page individually. We also collected information on the type of device, browser used, and the traffic source (ie, an organic search in a search engine, directly typing in the Web site URL, or links from either a referral Web site, social media, or e-mail).

We collected Web site metrics on the day each dissemination activity was delivered. The data were described using means and percentages. Chi-square test for trend was performed to evaluate year-over-year use data. All statistical analysis was conducted using Stata 11.0.^17^ We obtained ethics approval from the University of Calgary Conjoint Health Research Ethics Board (REB13-0729).

### Dissemination Activities

Dissemination activities were grouped into 4 categories based on the mode of knowledge delivery: print materials, electronic materials, in-person meetings, and a laboratory prompt with a hyperlink to the CKD-P. For each instance of a dissemination activity that occurred from launch day, November 5, 2014, to May 31, 2019, we recorded the date.

#### Print

Printed materials are a simple and cost-effective medium to disseminate knowledge.^18^ The printed materials included faxes sent to all primary care physicians in the province of Alberta, Canada, along with posters and brochures that were displayed or handed out during conferences and training sessions. The material included a short description of the CKD-P (purpose and content) and the Web site address.

#### Electronic

For electronic dissemination, we primarily targeted relevant professional associations’ electronic newsletters and also disseminated information via social media (Twitter). We collaborated with academic institutions and networks such as Alberta Health Services (AHS), the Interdisciplinary Chronic Disease Collaboration (ICDC), the Alberta Kidney Disease Network (AKDN), and the Universities of Calgary and Alberta to disseminate electronic newsletters, e-mails, Web site posts, live podcasts, and video conferences.

#### In-person meetings (categorized as small and large)

These meetings provided forums for primary care providers to learn how to use the CKD-P.

##### Small

We presented the CKD-P at small in-person meetings, defined as meetings with 50 or fewer individuals attending. They included medical trainee workshops, accredited continuing medical education sessions, city-wide renal rounds, evening courses, and research group rounds.

##### Large

Presentations were also provided at large in-person meetings that had greater than 50 individuals attending. These included the Annual Family Practice Review Conference, Accelerating Primary Care Conference, and Canadian Society of Nephrology presentations. Most large in-person meetings occurred in Canada, but some occurred internationally, such as in the United States, Indonesia, and India.

#### Electronic medical record laboratory prompt

We also worked with provincial laboratories and the most commonly used primary care electronic medical record vendor used in Alberta (TELUS Health) to create a laboratory prompt. The prompt included a point-of-care hyperlink to the CKD-P *diagnose* page that accompanied abnormal eGFR lab results (eGFR < 60 mL/min/1.73 m^2^). This prompt became available in November 2016.

## Results

### User Overview

From November 5, 2014, to May 31, 2019, there were a total of 83 294 users, 90 805 sessions, and 231 684 pageviews on the CKD-P Web site. There were 59 604 new users. The average number of sessions per user was 1.5, and they accessed on average 2.6 pages during each session. The average session duration was 2 minutes and 8 seconds and the Web site bounce rate was 45.7%. In all, 74% of users were from Canada, 12% from Indonesia, and 4% from the United States. Within Canada, a majority of the users were located in Calgary, Alberta (30.7%), followed by Edmonton, Alberta (11.0%), and Toronto, Ontario (4.3%).

Chrome, Internet Explorer, and Safari were the most common browsers used to access the CKD-P, with the proportion of sessions being 39.3%, 24.2%, and 17.9%, respectively. The Web site was equally accessed using organic searches (48.5%) or by directly typing in the URL (42.7%). Less than 10% of the remaining sessions were initiated from a referral source (ie, a link from social media or e-mail). When comparing the first (2014-2015) and last (2018-2019) years of dissemination, mode of Web site access was consistent over time, primarily from a desktop computer (76% vs 78%). This was followed by Web site access using a mobile (17% vs 18%) and then a tablet (7% vs 4%).

### Google Analytics Metric Trends

**Figure 1.**
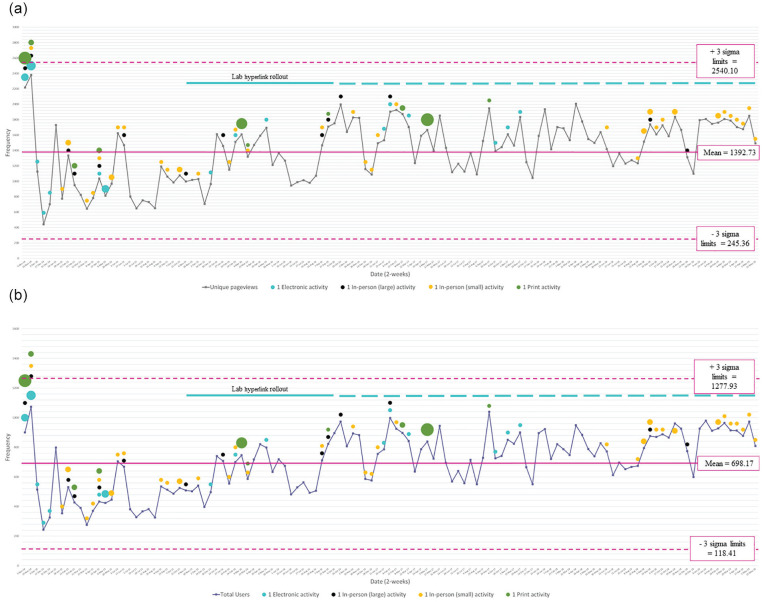
Control charts of Google Analytics metrics and dissemination activities for the CKD pathway over time: (a) unique pageviews and (b) total users. *Note.* The size of the dot is representative of the number of activities disseminated. The dots shown in the legend represent one disseminated activity. The largest green dot seen on March 3, 2017, represents 6 print activities. The blue solid line represents the period that the lab hyperlink was actively rolled out. The means were calculated as the average of the plotted number of unique pageviews or total users.

We found statistically significant increasing trends in specific Google Analytics metrics over time (Table 1). The total users and new users almost doubled from 2014 to 2019 (*P* for trend = .008 and .009, respectively) and the number of sessions per day increased almost 2-fold (*P* for trend = .006). An increase in the number of pageviews per day was also observed (106.7 pageviews/day to 162.6 pageviews/day; *P* for trend = .016). The mean number of unique pageviews was 1392.73, the positive 3 sigma limit was 2540.10 pageviews, and the negative 3 sigma limit was 245.36 pageviews (Figure 1a). The mean total number of users was 698.17 users, the positive 3 sigma limit was 1277.93 users, and the negative 3 sigma limit was 118.41 users (Figure 1b). We observed a shift toward an increasing number of unique pageviews and total number of users overtime. Web site traffic appeared to be maintained above the means during the last year of dissemination.

**Table 1.** The Number of Dissemination Activities and Google Analytic Metrics Overall and Over 1-Year Periods.

	Overall (N = 1669 days)	Nov 5, 2014-Nov 4, 2015 (N = 365 days)	Nov 5, 2015-Nov 3, 2016 (N = 365 days)	Nov 4, 2016-Nov 3, 2017 (N = 365 days)	Nov 4, 2017-Nov 3, 2018 (N = 365 days)	Nov 4, 2018-May 31, 2019 (N = 209 days)	*P* value for trend
Number of dissemination activities							
Print (n)	28	12	8	8	0	0	
Electronic (n)	23	14	3	6	0	0	
In-person large (n)	17	8	5	1	2	1	
In-person small (n)	37	14	5	3	11	4	
Google Analytics metrics							
Average total users per day	35.7	25.3	34.1	40.4	40.6	47.6	.008
Average new users per day	35.7	25.1	33.3	39.4	39.8	44.9	.009
Average sessions per day	54.4	38.9	50.8	59.3	60.6	68.7	.006
Average pageviews per day	138.8	106.7	131.1	151.9	152.1	162.6	.016
Average number of sessions per user	1.5	1.5	1.5	1.5	1.5	1.4	
Average pages per session	2.6	2.7	2.6	2.6	2.5	2.4	
Average session duration (hour:minute:second)	0:02:08	0:02:31	0:02:10	0:02:06	0:02:03	0:01:52	
Average bounce rate (%)	45.7	47.3	46.7	43.2	44.3	48.6	

### User Behavior

**Figure 2.**
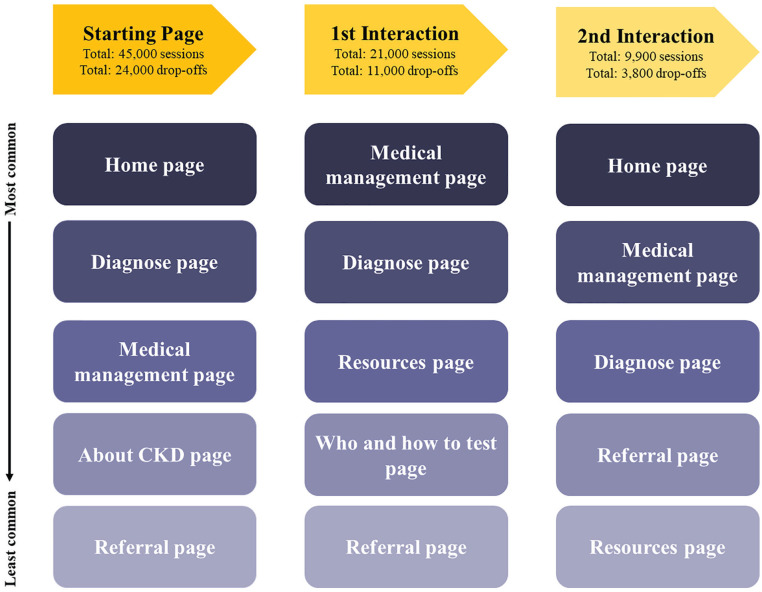
Google Analytics user flow diagram of the starting pages and following interactions. *Note.* The starting page is the first page users begin their session with on the CKD-P Web site. The most common starting page was the CKD-P home page, followed by the diagnose page, medical management page and so on. The first interaction is the next page that users may go to after leaving the starting page. The second interaction is the page that users may go to after leaving their first interaction page. CKD = chronic kidney disease.

When reviewing behavior data over the most recent 2 years (June 1, 2017-May 31, 2019) (Figure 2), the *home* page was the most popular starting page on the Web site with 29 644 (65.8%) sessions and 31 391 (39.2%) unique pageviews (Table 2). Thereafter, approximately 8400 (18.7%) sessions proceeded to the *medical management* page and 5400 (12.0%) sessions proceeded to the *diagnose* page. The bounce rate for the *home* page (39.6%) was relatively low compared to the bounce rates for the *medical management* and *aboutckd* pages: 73.5% and 89.4%, respectively. The average time spent and the percentage of exits on the *medical management* and *aboutckd* pages were approximately double the time percentage of exits on the *home* and *diagnose* pages.

**Table 2.** User Behavior by Web site Page From June 1, 2017, to May 31, 2019.

Page	Entrances	Bounce rate (%)	Pageviews	Unique pageviews	Average time on page (second)	% exit
Home	29 644	39.6	40 304	31 391	64.2	37.7
Diagnose	5851	28.2	27 932	13 694	66.8	30.6
Medical management	4198	73.5	19 926	16 089	132.1	56.0
About CKD	2487	89.4	4034	3602	166.4	69.3
About the pathway	953	67.7	6219	5055	105.2	42.3
Referral	693	34.1	2486	2183	36.6	25.7
Resources	678	73.0	5620	4757	110.7	50.9
About, who, and how to test	358	62.3	3609	2935	94.4	28.2
Index	120	100.0	120	120	0.0	100.0
Contact	45	51.1	547	318	58.9	15.0

CKD = chronic kidney disease.

### Dissemination Activities

Throughout the 5-year period, we disseminated 28 print activities, 23 electronic activities, and 54 in-person activities (Table 3). Of the in-person activities, 37 were small-scale meetings and 17 were large-scale meetings. Our most frequently disseminated print and electronic materials were faxes and electronic newsletters, respectively. Dissemination activity was most intensive during the first year, when all dissemination methods were used relatively equally (Figure 1). Over time, the number of dissemination activities decreased. In-person meetings and the laboratory prompt were the only activities delivered during the last 2 years. We were unable to capture Google Analytics metrics for visits directly related to traffic from the laboratory prompt because our analytics settings were not configured to track specific source pages. As the laboratory prompt linked users directly to the *diagnose* page as opposed to *home* page (which was used on all other dissemination material), visits that had the *diagnose* page as a starting page may be related to the laboratory prompt. During this time, the total number of users and unique pageviews appeared to be relatively constant around 1760 unique pageviews and 900 total users per 2-week period, respectively.

**Table 3.** Number and Type of Dissemination Activities Over a 5-Year Period.

Dissemination activities	N
Print	28
Posters	4
Brochures	10
Fax (CPSA list)	14 days
Electronic	23
Newsletters	12
Mass media (TV, social media)	4
Web site post	2
E-mail	1
Live podcast	3
Video conference	1
In-person	54
Small-scale meeting (≤50 people)	37
Large-scale meeting (>50 people)	17
Laboratory prompt	30 months

CPSA = College of Physicians and Surgeons of Alberta.

When we measured Web site traffic on the day the dissemination activities occurred, we found that the number of users increased by 2-fold 42% (44/105) of the time and by 3-fold 12% (13/105) of the time. (Table 4). Similarly, our methods demonstrated a 2-fold and 3-fold increase in daily pageviews 21% (22/105) and 12% (13/105) of the time, respectively. Each dissemination method (print, electronic, and in-person meetings) appeared to have a similar proportional increase in daily users and pageviews.

**Table 4.** Activity Percentage Associated With a 2- or 3-Fold Users or Pageview Increase From Overall Mean.

Dissemination activity	Total number of activities (N = 105)	Daily users		Pageviews	
		Number of activities on the day there was a 2-fold increase, n (%) (N = 44)	Number of activities on the day there was a 3-fold increase, n (%) (N = 13)	Number of activities on the day there was a 2-fold increase, n (%) (N = 22)	Number of activities on the day there was a 3-fold increase, n (%) (N = 13)
Print	28	9 (32.14%)	2 (7.14%)	4 (14.29%)	2 (7.14%)
Electronic	23	11 (47.83%)	4 (17.39%)	6 (26.09%)	4 (17.39%)
Small in-person	37	17 (45.95%)	5 (13.51%)	8 (21.62%)	5 (13.51%)
Large in-person	17	7 (41.18%)	2 (11.76%)	4 (23.53%)	2 (11.76%)

## Discussion

### Key Findings

We used Google Analytics to describe the reach and use of the CKD-P using a multi-faceted dissemination strategy over a 5-year period. There were significant positive trends in the use of the CKD-P Web site among new and returning users overtime. While we primarily disseminated the CKD-P through in-person meetings, followed by print and electronic activities, we found that it was challenging to differentiate the impact of each dissemination activity in promoting the use of this clinical pathway tool using Google Analytics. Repeated multi-faceted dissemination of activities over time appeared to play a role in increasing the reach and use of the CKD-P Web site.

When designing and implementing an online clinical pathway tool, it is essential to understand if the needs of the target audience are met. This can be explored by identifying the source of traffic and the type of device used to access and use the Web site. We found that the CKD-P Web site was primarily accessed using a desktop with Chrome or Internet Explorer browsers by either an organic (via a search engine) or direct (via the Web site URL link) search. This highlights that the Web site was being viewed on the device the pathway was designed for, which was based on preferences identified by primary care providers in a previous implementation study for the CKD-P tool.^19^ Accessing the Web site using an organic search suggests that users were intentionally looking for the CKD-P tool. In addition, as the Web site was equally accessed using direct searches, providing the CKD-P Web site URL link in electronic dissemination activities appeared to have played a role in driving Web site use. Google Analytics behavior data can also provide insight into whether the needs of the audience are met. For example, the most popular starting page on the Web site was the *home* page, followed by the *diagnose* page. As the CKD-P laboratory report prompt links health care providers directly to the *diagnose* page when abnormal eGFR results are reported, the popularity of this starting page may reflect successful implementation of the laboratory prompt in primary care electronic medical records. Furthermore, the *medical management* page was the most popular first interaction page. The *medical management* page average session duration was 2 times longer than the *home* page, suggesting that users read the information about lifestyle changes and drug therapy on this page. Our Web site metrics data suggest that users of the CKD-P tool are investing time into learning about current guidelines on the diagnosis and management of patients with CKD. This is encouraging given the high prevalence of individuals living with unrecognized CKD in Canada.^20^

To ensure that long-term, up-to-date guidance and supporting information for primary care providers who care for adults with CKD is readily available, the sustainability of the CKD-P tool and our approach to knowledge dissemination should be considered. For instance, bounce rate is a key indicator of user satisfaction and how engaging a Web site is.^21^ While levels to define a “good” or “bad” bounce rate have not been defined in the health literature, in the gray literature such as business blogs, a bounce rate of 40% to 55% is defined as good.^22^ The first impression of a Web site has been shown to have a lasting impact in determining whether users return to the Web site; a lower bounce rate is correlated with an increased return visit rate.^23^ The CKD-P average bounce rate was 45.7% over the study period and is similar to other health-related web traffic studies.^23-26^

Understanding the dissemination methods that effectively reach the target audience may be a greater driver for success in sustaining overall Web site traffic than the number of activities disseminated. Among our target audience of primary care providers, print materials and interactive small group discussions were previously identified as useful tools for sharing knowledge.^8,27^ A systematic review also found that educational outreach visits and meetings were the most effective strategies for managing knowledge to inform clinical decision making in primary care when compared to passive dissemination strategies such as distributing educational materials.^28^ In our study, the most frequent dissemination activity was in-person meetings, and over 90% of new users came from direct or organic searches rather than links embedded in electronic materials. Our data also showed that during the final year of dissemination where only in-person activities were disseminated, the number of unique pageviews and the total number of users appeared to be sustained at levels that were higher than the mean. This suggests that in accordance to the literature in the primary care setting, in-person meetings may have played a role in increasing the awareness of the CKD-P, when compared to electronic or print disseminations. In addition, as the laboratory prompt leads health care providers to the *diagnose* page on the CKD-P Web site, the number of pageviews on this page may provide insight on the usefulness of this dissemination activity. Considering that the *diagnose* page was the second most popular starting page with the lowest bounce rate, this suggests that clinicians had access to the pathway at the point of care and were engaging with the Web site. Although few studies have used embedded laboratory prompts to improve awareness and access to clinical pathway tools,^29^ it appears that the prompt was a suitable way to reach our target audience.

Compared to health-related studies that implemented a single approach to dissemination,^24,25^ it appears that our ongoing dissemination approach using repeated delivery of in-person activities and the use of embedded links in existing health platforms promoted the use and reach of the tool. The limited number of studies that have used Google Analytics to evaluate health-related Web site uptake also found that holding large-scale campaigns for longer periods of time can be beneficial in sustaining the number of visits to a Web site, particularly when information is spread using word-of-mouth.^13^

### Limitations

There are limitations to our study that should be considered while interpreting the results. First, the data acquired using Google Analytics are’ a proxy measure of the relationship between a dissemination activity and changes in CKD-P user traffic. We assumed that changes in Google Analytics metrics were indicative of a relationship between a dissemination activity and CKD-P use. However, dissemination activity categories often co-occurred, and we were not able to tease out individual drivers of Web site uptake. This was a limitation when assessing the use of the laboratory prompt, but we used the number of pageviews on the *diagnose* page as a proxy measure of Web site traffic potentially related to this dissemination strategy. Second, although our pathway targets primary care providers, we were not able to confirm if all users were primary care providers and if word-of-mouth dissemination among providers/researchers drove people to use the CKD-P. As a result, we are not able to directly assess the association of the CKD-P with outcomes such as appropriateness of nephrology referrals. We have, however, recently reported significantly higher rates of albumin-to-creatinine ratio (ACR) testing and prescription of guideline-recommended angiotensin-converting enzyme inhibitors, angiotensin II receptor blockers, and statins in regions where the CKD-P was most actively used.^30^ Increased CKD-P awareness among health care providers may have contributed to improvements in CKD patient management. Third, as new users are tracked using cookies, if they erase their cookies and return to the Web site, they would be considered a new user. This may result in an overestimation of the number of new users. Similarly, if different users access the Web site using the same device (ie, a shared monitor in a clinic), only one user would be recorded in Google Analytics, resulting in an underestimation of new users. These are limitations inherent to using Google Analytics. Future work will look at clinical outcomes and experiences of users of the CKD-P.

## Conclusion

In this retrospective descriptive study, we used Google Analytics to describe the reach and use of the CKD-P tool after conducting various dissemination activities. It was challenging to differentiate the impact of each dissemination activity. In-person activities and a laboratory prompt embedded in primary care electronic medical records appeared to play a role in increasing uptake. Future studies describing how dissemination activities are related to clinical pathway utilization in primary care environments are needed to better understand and optimize the interest and uptake of online clinical tools among primary care providers.
